# A Pulsatile Bioreactor for Conditioning of Tissue-Engineered Cardiovascular Constructs under Endoscopic Visualization

**DOI:** 10.3390/jfb3030480

**Published:** 2012-07-19

**Authors:** Fabian König, Trixi Hollweck, Stefan Pfeifer, Bruno Reichart, Erich Wintermantel, Christian Hagl, Bassil Akra

**Affiliations:** 1 Chair of Medical Engineering, Technical University Munich, Boltzmannstrasse 15, Garching 85748, Germany; Email: f.koenig@mytum.de (F.K.); stefan.pfeifer@tum.de (S.P.); wintermantel@tum.de (E.W.); 2 Department of Cardiac Surgery, Medical Center Munich University, Marchioninistr. 15, Munich 81377, Germany; Email: Trixi.Hollweck@med.uni-muenchen.de (T.H.); Bruno.Reichart@med.uni-muenchen.de (B.R.); Christian.Hagl@med.uni-muenchen.de (C.H.)

**Keywords:** bioreactor, tissue engineering, dynamic cell conditioning, heart valve disease

## Abstract

Heart valve disease (HVD) is a globally increasing problem and accounts for thousands of deaths yearly. Currently end-stage HVD can only be treated by total valve replacement, however with major drawbacks. To overcome the limitations of conventional substitutes, a new clinical approach based on cell colonization of artificially manufactured heart valves has been developed. Even though this attempt seems promising, a confluent and stable cell layer has not yet been achieved due to the high stresses present in this area of the human heart. This study describes a bioreactor with a new approach to cell conditioning of tissue engineered heart valves. The bioreactor provides a low pulsatile flow that grants the correct opening and closing of the valve without high shear stresses. The flow rate can be regulated allowing a steady and sensitive conditioning process. Furthermore, the correct functioning of the valve can be monitored by endoscope surveillance in real-time. The tubeless and modular design allows an accurate, simple and faultless assembly of the reactor in a laminar flow chamber. It can be concluded that the bioreactor provides a strong tool for dynamic pre-conditioning and monitoring of colonized heart valve prostheses physiologically exposed to shear stress.

## 1. Introduction

The incidence and prevalence of heart valve disease is increasing worldwide. Up to 300,000 heart valves [1] are replaced each year and although valve repair is currently the preferred method of treating patients with severe heart disease, the predominant treatment for end-stage valvular heart disease is valve replacement [2]. As cardiac tissue is highly specialized and, in contrast to striated muscles such as biceps and quadriceps, not capable of repairing itself, valve replacement is often the only option [2,3]. At present, three types of heart valves are in clinical use: mechanical, biological and homograft and each type has severe limitations [4]. The consequences are severe, especially for pediatric patients. Approximately 2% of infants suffer from heart valve disease and therefore have to undergo multiple surgical or interventional procedures to restore the functionality of the replaced or reconstructed vessel [5]. A new approach to a better solution is made by tissue engineering. Tissue engineering combines the principles and methods of engineering and life science for the development of biological substitutes to restore, maintain or improve tissue functions [6]. The desirable characteristics of a heart valve grown *in vitro* would basically be a stable geometry with a potential for growth and regeneration within the patient [3]. There are two possibilities to profit from the advantages of tissue engineering. Either the whole valve can be engineered from human cells or a scaffold can be seeded with cell layers [2]. The creation of the whole valve has never shown promising results so far [7]. First, the necessary stability to withstand the high stresses cannot be reached so far. Secondly, the cultivation of such large amounts of cells needed has not been accomplished yet. Therefore research is mainly focusing on cell seeding of synthetic valves at the moment. Depending whether pediatric or adult patient should be treated, several scaffold materials are under investigation. Synthetic degradable polymers like polyglycolic acid (PGA) or polylactid acid (PLA) undergo degradation after implantation while an ECM is formed by colonized cells [8], which seems beneficial for growing pediatric patients. However, degradable polymers could denature in rates not matching those for tissue formation and release degradation products which affect biocompatibility [9]. Synthetic, non-degradable polymers like polyurethane (PU) are mainly characterized by their structural resistance, non-immunogenic and anti-thrombotic properties [10] favorable for the therapy of adult patients. However, a large challenge is the creation of a stable and confluent endothelial layer on valve scaffolds [3]. To enable the proliferation and differentiation of highly complicated cell structures like the endothelium, the cells have to be cultivated in bioreactors. The general idea is to provide a similar environment, including not only pH, temperature and other “classical” factors, but also stress exposure [11]. In the human body, cells are permanently subjected to and stimulated by mechanical, electrical, and chemical signals and gradients that influence their behavior, phenotype, shape, properties, and the proliferation rate [12]. Bioreactors represent the ideal possibility to study such effects on the generation of tissues. It has been found, that, if these signals are absent or improperly chosen, cells only poorly proliferate and cannot form organized tissues [13]. The cells dedifferentiate and no extracellular matrix (ECM) is established. To achieve a good quality ECM, mechanical stress is essential [12]. Therefore, specific bioreactors are indispensable for creation and regeneration of complex 3D tissue structures. Another valuable tool of a bioreactor is the possibility of perfusion. By perfusing culture medium directly through the pores of a cell-seeded 3D scaffold, thereby reducing mass transfer limitations both at the construct periphery and within its internal pores, metabolites can be transported into deeper cell-layers and cell survival, growth and function can be drastically enhanced. In conclusion, supplying metabolites, removing catabolites, maintaining temperature, establishing and monitoring pH, applying mechanical stresses that stimulate the formation of the ECM, and allowing cohesion between cells are the main functions that a bioreactor has to provide [14]. The aim of this study was to develop a new bioreactor which simulates physiological conditions and provides the possibility to gradually increase shear stress induced to the endothelial layer.

## 2. Results and Discussion

### 2.1. Bioreactor Assembly

**Figure 1:**
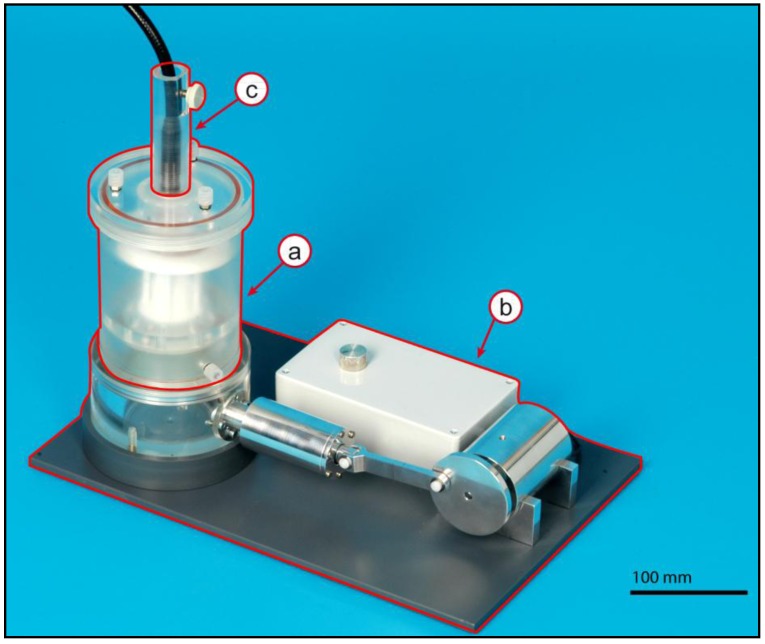
Bioreactor assembly: The bioreactor-system can be divided in the core unit with TS (**a**); the actuation unit (**b**) and the monitoring unit (**c**) Scale bar = 100 mm.

As shown in Figure 1, the pulsatile conditioning bioreactor consists of a core unit with Teflon^®^ support (TS) (a); an actuation unit (b) and a monitoring unit (c) built from several assembly groups.

The bioreactor was designed to precondition polyurethane heart valve (PUHV). Earlier approaches attempted to create artificial heart valves by statically seeding of autologous cells on PU heart valves followed by shear stress exposure. Due to this sudden change of environment, the cells are not able to adapt to the new conditions and are lost by flow rates far below physiologic conditions. Numerous bioreactors for conditioning of heart valves by flow are previously described [13,15,16,17,18,19]. However, these bioreactors have major drawbacks. All these systems have a bulky design [20] and work with a lot of tubes [13,21] and screws [3]. In contrast, the bioreactor presented in this study has a compact and clearly arranged design and avoids the use of screws in the sterilized parts for an easy assembly of the bioreactor. Due to the need for sterility, this bioreactor was designed to be assembled in a laminar flow cabinet without any further tools like screwdrivers, *etc.*

### 2.2. Core Unit

As mentioned before, another drawback of existing bioreactors is tubing. The usage of tubes is a huge expense factor. Since the tubes have to be sterile, disposable tubes have to be used and have to be changed after every conditioning phase, which leads to high running costs. The second and more severe disadvantage of tubes is that they do not have any junctions. The lack of junctions leads to complicated methods of fixing and is immensely increasing the risk of infection. Furthermore, the fixing of the tubes demands the use of special tools again. Our bioreactor completely relinquishes tubing. While this may lead to slightly higher immediate nonrecurring manufacturing costs, the running costs as well as the risk of infection are minimized. 

**Figure 2:**
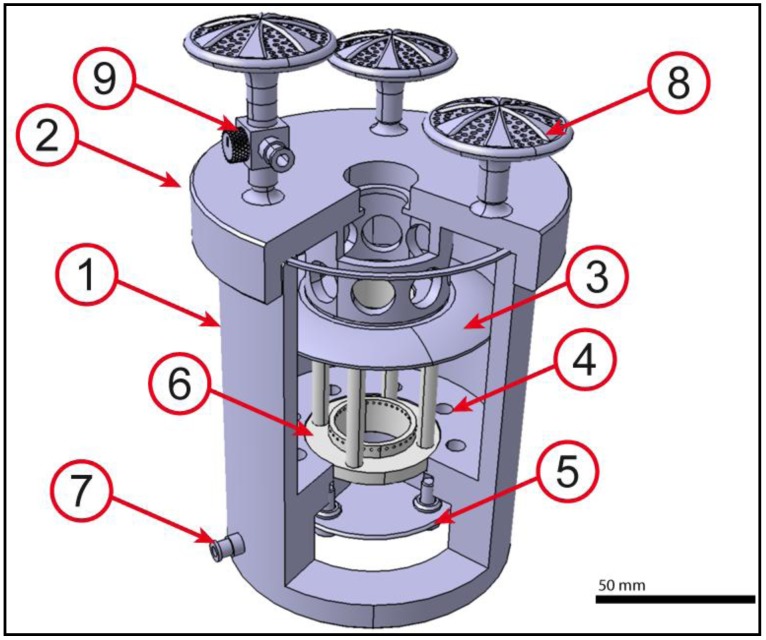
Core unit of the bioreactor: Casing (**1**); lid (**2**); disc (**3**); separation plate (**4**); valve (**5**); TS (**6**); cleanout (**7**); gas filters (**8**); three-way stopcock (**9**). The core unit contains the TS, provides a circulating flow through the PUHV and allows monitoring and exchange of culture medium. Scale bar = 50 mm.

The core unit (Figure 2) is the central part of the reactor and provides a stable retaining fixture, in which the TS can be fixed to keep PUHV orientation upwards, meaning that the leaflets open upwards. The fluid is channeled upwards through the valve and circulates back through the outside of the valve. The main task of the valve-leaflets is to prevent any backflow through the valve. Therefore a possibility for the recirculation of the medium is provided. Moreover it is granted that the flow through the valve (the up-flow) is strong enough to open the valves correctly. The flow is channeled to ensure sufficient pressure and flow volume. The reactor further offers medium cleanout by a luer-lock-connector during the process to avoid cell damage due to flow break. This connector can also be used for pressure measurements. Manufacturing of the core unit from acrylic glass provides optical transparency for macroscopic observation of processes within the unit. Physiological conditions (37 °C/5% CO_2_) are provided by integrating the bioreactor in a standard incubator. Sterile filters connected by luer-locks to the lid secure CO_2_-exchange. On one luer-lock a three-way stopcock is interposed to allow medium and cell addition. 

**Figure 3:**
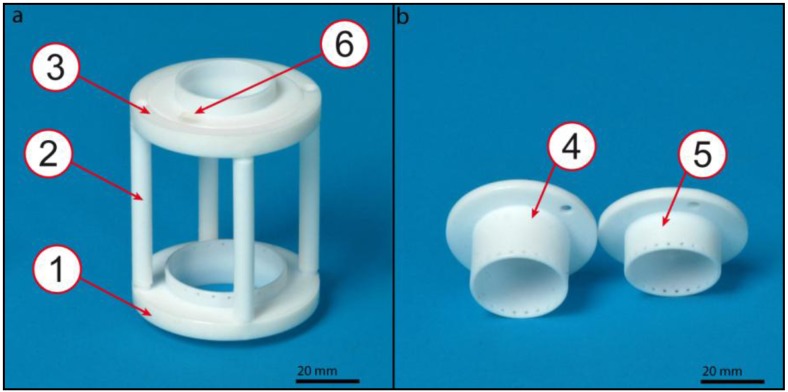
Teflon^®^ support (TS) for heart valve fixation: The TS (**a**) consists of a bottom plate (1), four bars (2) and a lid (3). The lid can be assembled by a screw (6) with three different inserts (**b**) to vary the distance between the valves fixing points (4,5) while maintaining a constant length of the TS. Scale bars = 20 mm.

The TS (Figure 3), consisting of a bottom plate, a lid with variable inserts and four connecting bars, was designed to avoid heart valve deformation during conditioning and to compensate loss of height after sampling.

### 2.3. Actuation Unit

**Figure 4:**
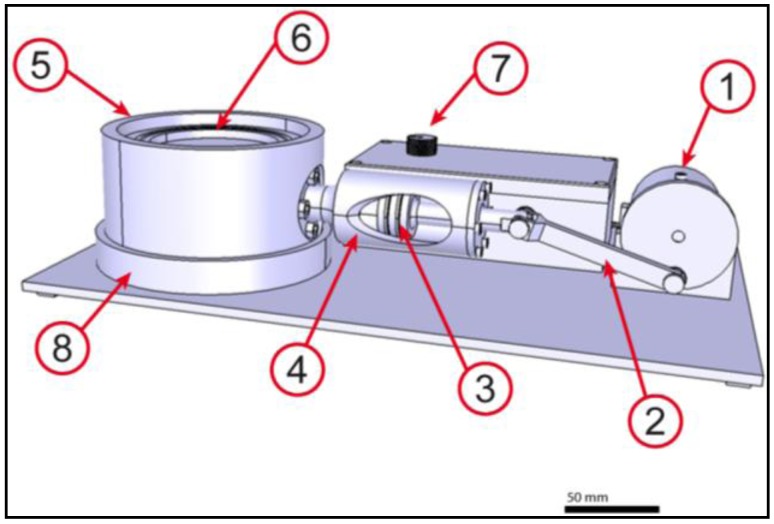
Actuation unit of the bioreactor: Motor connected to an eccentric (**1**); connection rod (**2**); piston (**3**); cylinder (**4**); air chamber (**5**); fixing coil (**6**); speed regulator (**7**); base plate (**8**). The actuation unit generates a pulsatile flow and provides a compact and stable base for the whole bioreactor system. The flow is adjustable from one to sixty pulses per minute. Scale bar = 50 mm.

The actuation unit (Figure 4) is the most complex part of the bioreactor and provides a pulsatile flow for the core unit. Therefore, a special engine was designed. A speed regulator (7) is connected to the motor (1) for a continuously alignment of 0–60 rpm. The motor turns the cam (2) which is fixed to the piston rod (3). The piston rod is conducted in the cylinder (4) which leads to a horizontal movement of the piston in the cylinder. The cylinder is connected to the air chamber (5) of the bioreactor, which mainly consists of an air chamber and a membrane (6) (free movement: d = 70 mm), balancing displaced volume. Calculated flow constitutes 31,500 mm^3^ per cycle, which adds up to about 2 L/min. To avoid membrane overstretching, a flow of 31,000 mm^3^ (≈1.86 L/min) was applied.

### 2.4. Monitoring Unit

The complicate process of conditioning heart valves requires monitoring and maintaining the correct framework conditions. Temperature, pH and CO_2_ concentration have to be controlled as well as the correct opening and closing of heart valve leaflets. While previous reactors controlled temperature, CO_2_ and the cellular growth medium [17], options to monitor the correct functioning of the valve have not been provided. Because our bioreactor is compact enough to fit into a standard incubator, temperature and CO_2_ concentration do not have to be controlled by the bioreactor itself. 

**Figure 5:**
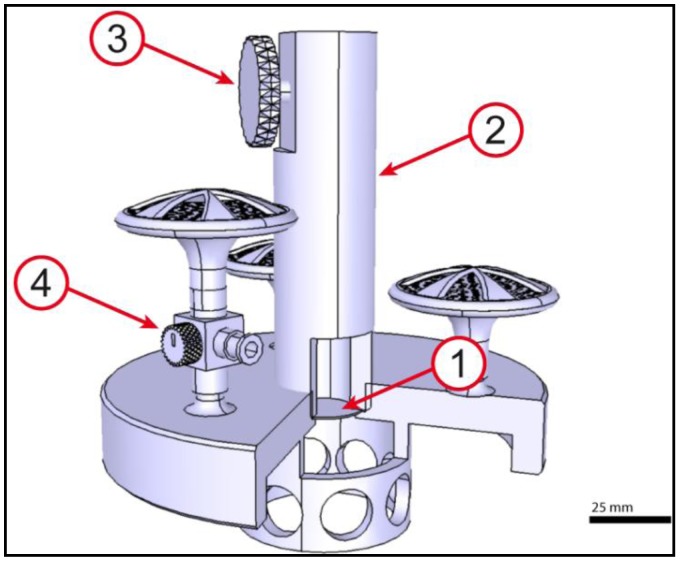
Monitoring unit of the bioreactor: Glass plate (**1**); cylinder (**2**); fixing screw (**3**); luer-lock with attached three-way stopcock (**4**). The monitoring unit grants a stable and secure way to observe the PUHV without risk of contamination. A seal ring on top of the glass plate ensures a sealed off environment inside the bioreactor while providing a clear view on the valve leaflets. The endoscope can be secured with the fixing screw. Scale bar = 50 mm.

Moreover, the monitoring unit (Figure 5) of our bioreactor allows heart valve observation with a technical endoscope. The endoscope is strictly isolated from the sterile core unit to keep the risk of infection to a minimum. Two concentric holes with different diameters in the cap enable the insertion of a thin glass plate (1). The glass plate is pressed against the rim by a cylinder (2) which is screwed into the cap. The cylinder locks the glass plate into position and also serves as an endoscope fixing. A gasket ring is attached at the bottom of the cylinder, to protect the glass plate and to enable a maximal contact pressure. The endoscope can be fixed in the cylinder by a retaining screw (3) to ensure that it is in a locked position. The attached three-way stopcock (4) allows ingestion of culture medium or cell suspension.

### 2.5. Bioreactor Function

A pulsatile flow is created in the core unit without any fluid exchange between the core unit and the actuation unit as well as without any contact of the medium with unsterile parts.

**Figure 6:**
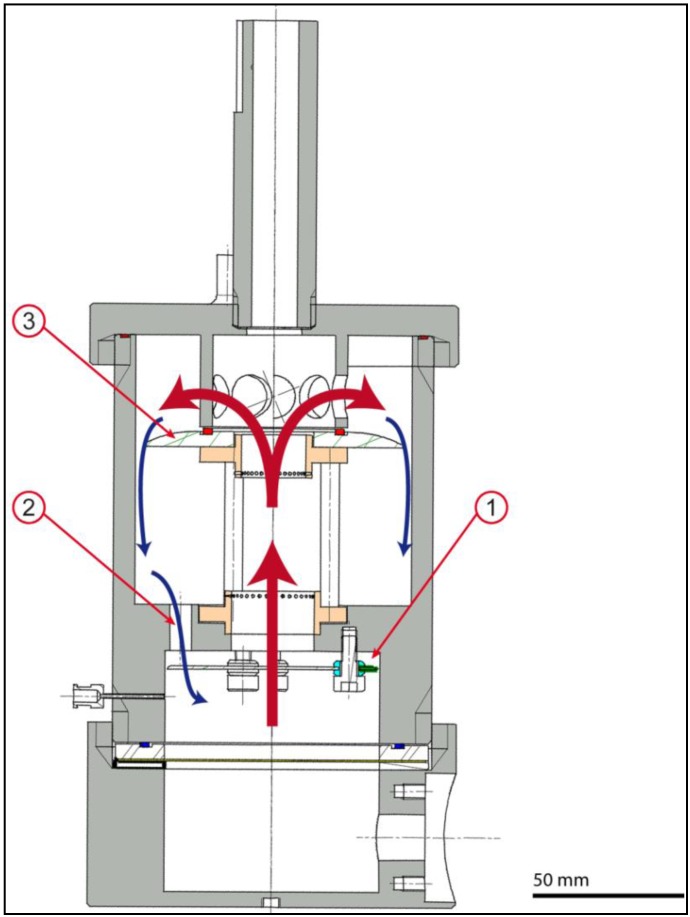
Flow circulation: During the up-flow, the valve (**1**) is pushed upwards and seals the small holes (**2**). Culture medium flows upwards through the valve and backwards through the small holes. A backflow through the valve is prevented by PUHVs leaflets. The disc (**3**) prevents medium backflow along the external wall of the PUHV. Scale bar = 50 mm.

The bioreactor activity is controlled and regulated by the speed regulator adjusting motor velocity. The motor is driving the extender wheel which transfers the rotation into a linear movement of the piston. The piston increases the pressure in the air chamber during the forward movement and decreases it while moving backwards. Consequently, the membrane is moved up and down each cycle, resulting in a flow (Figure 6) of culture media in the upper chamber. To overcome the problem of flow channeling, the bioreactor is equipped with a valve (1), working in opposite direction of the PUHV. Every time the actuation membrane is curved upwards, the medium flow moves the membrane against the separating plate, resulting in a closure of the eight smaller holes (2). The whole flow is directed through the main flow drill hole (MFDH) and passes the PUHV at opened leaflets. When the actuation membrane is lowered again, the pressure in the lower chamber of the core unit decreases and the up-flow stops. The leaflets seal the heart valve, the membrane glides back into its resting position and the medium flows back through the eight smaller holes. The disc (3) directs the flow of the medium along the casing wall and avoids a medium flow along the external wall of the PUHV, preventing cellular coverage from mechanical stress.

The medium runs upwards through the MFDH in the center and streams back through the smaller holes. To provide a stable retaining fixture for the TS, the MFDH has an offset so that the TS can be slid into the CU. A second offset was added, to grant a constant diameter in the MFDH to avoid any turbulence. Due to this form closure, the TS is horizontally located into position. The TS is clamped by the cap of the reactor which is screwed on top of the casing and thereby arrests the cage in vertical direction. Between the TS and the cap a disc is integrated. On the top of the disc, a joint ring is arranged, which dampens the tension and thereby protects the Teflon support.

### 2.6. Bioreactor Sterility

Culture medium was checked during the bioreactor run at 5 days and 10 days for signs of contamination by conventional streak samples. Medium was examined for aerobic growth, particularly pathogenic saccharomyces or blastomycetes. After 15 days of observation, the air chamber was checked for fluids. Microbiological analysis and macroscopically inspection showed negative results, indicating sterile operation conditions if handled correctly.

### 2.7. Bioreactor Functionality

Appliance, function and handling of the bioreactor were tested and showed the desired results. The whole bioreactor system worked properly and in a satisfactory manner. The simple and tubeless design led to a great acceptance of the bioreactor by the users. The complete omission of tubes leads to a failsafe design of the bioreactor. All components of this bioreactor can only be assembled in one specific and clear way. The risk of reverse connections and the resulting destruction of the heart valves are eliminated in this design since there is only one possible way to assemble the bioreactor. However, most of the state-of-the-art bioreactors require screws and tubes for assembly. This increases the required time and complexity for assembly as well as the risk of errors [22,23,24,25,26,27,28,29,30]. The compact design and mounting on a baseplate of our bioreactor grants stability during preconditioning and allows an easy and quick handling and transportation. Other bioreactors found in literature consist of many loose components without any stabilizing structure [22,23,24,25,29].

As a result of our assembly, the endoscope has clear vision of the upper side of the heart valve and the correct opening and closing of the valve leaflets can be accurately monitored. The endoscope can be connected to a computer via USB. Thus, no visual display or saving unit has to be attached to the bioreactor and the reactor design remains compact and easy to handle. The acquired data can be edited, saved or simply streamed on the computer. Visual control of the correct valve function is a helpful and important feature of this bioreactor. Most of previously described bioreactors are not equipped with a monitoring unit. With these bioreactors, monitoring is either only possible by visual inspection if the incubator is opened [27,28,30] or not at all [22,23,24,25,29].

**Figure 7:**
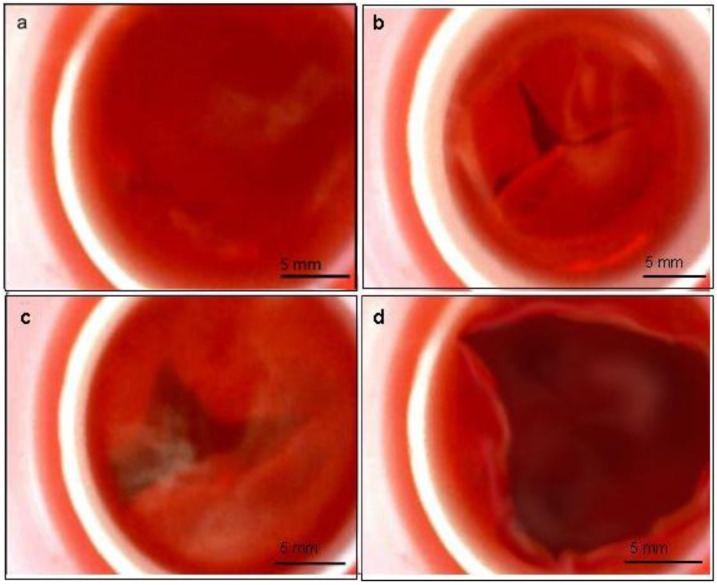
Assessment of bioreactor functionality by endoscopic observation: Functional efficiency of the bioreactor was demonstrated by perfect closure (**a**) and opening of the heart valve (**b**–**d**). Scale bars = 5 mm.

To ensure that the tissue engineered heart valve is preconditioned in a correct manner, the designed mechanism was examined. The bending of the membrane as well as the second valve worked as intended which resulted in the expected circling flow in the core unit. Testing the bioreactor with an unseeded PUHV showed that the pulsatile flow through the bioreactor is strong enough to induce a correct opening and closing of the valve leaflets (Figure 7). After quantification, an opening area of approximately 83% was determined. Thus, the designed actuation unit proofed sufficient for the preconditioning phase and no external pump is required. Most of the bioreactors found in literature rely on external actuators. Those require large tubes to connect the bioreactor inside the incubator with the actuation unit outside increasing the risk of contamination [22,23,24,25,26,27,28].

To evaluate the long-term behavior of the preconditioning system, the bioreactor was tested for 5 days. This survey showed that the bioreactor is capable of long-term loading. The motor withstood the high humidity and temperature in the incubator without any problems and showed no signs of fatigue. Likewise, the membrane withstood the high and continuous bending stresses. After 15 days of testing, visual examination of the membrane showed no signs of corrosion or rupture. The membrane should be exchanged preventively after three preconditioning cycles to avoid dynamic and elastic fatigue resulting in membrane rupture and consequently in contamination of the sterile compartment.

**Figure 8:**
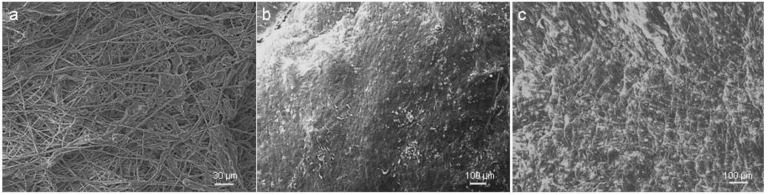
SEM analysis of seeded PUHV. Native (**a**) samples of PUHV showed disordered fibers resulting in a rough surface. PUHV revealed a confluent endothelial layer after seeding (**b**) with an EC alignment in flow direction after conditioning (**c**). Scale bars: a = 30 µm, b, c = 100 µm.

Another important difference between this bioreactor and the reactors found in literature [20] is that this bioreactor is not only designed for the actual conditioning process. This bioreactor was designed to be an intermediate step between the seeding and evaluation procedures. It allows slowly increasing the stress on the PUHV to grant the cells the possibility to adapt to shear stress and to develop an ECM. In this context, Ramaswamy *et al.* reported from a large collagen mass production after use of simulated pulmonary artery conditions using an organ-level heart valve bioreactor [22]. Zeltinger *et al.* described the stimulation of human dermal fibroblasts seeded onto a decellularized porcine matrix by a pneumatic flow bioreactor, resulting in the synthesis of ECM proteins [27]. For the fabrication of vascular grafts, Syedain *et al.* and Tschoeke *et al.* demonstrated the expression of collagen and other ECM components by human dermal fibroblast and ovine arterial myofibroblasts in fibrin gel [28,31]. However, complications such as thrombosis after the implantation of artificial grafts are caused in part by the lack of an intact endothelium [32]. To enable the development of an ECM and the formation of an endothelium, PUHV was consecutively seeded with fibroblasts (FB) and endothelial cells (EC) and were conditioned as described in the experimental section. Scanning electron microscopic (SEM) analysis (Figure 8) of native PUHV (a) demonstrated randomly orientated fibers resulting in a rough surface. PUHV showed a confluent cellular coverage prior conditioning (b) and after conditioning (c). In addition, cobblestone morphology indicates an intact endothelial layer after conditioning. Moreover, seeded PUHV exposed to flow revealed a cell alignment in flow direction. This is in contrast to results generated by Sierad *et al.* [26] demonstrating that EC did not completely cover all areas of the valve after seeding and perfusion at 600 mL/min for 17 d in a pulsatile conditioning system.

**Figure 9:**
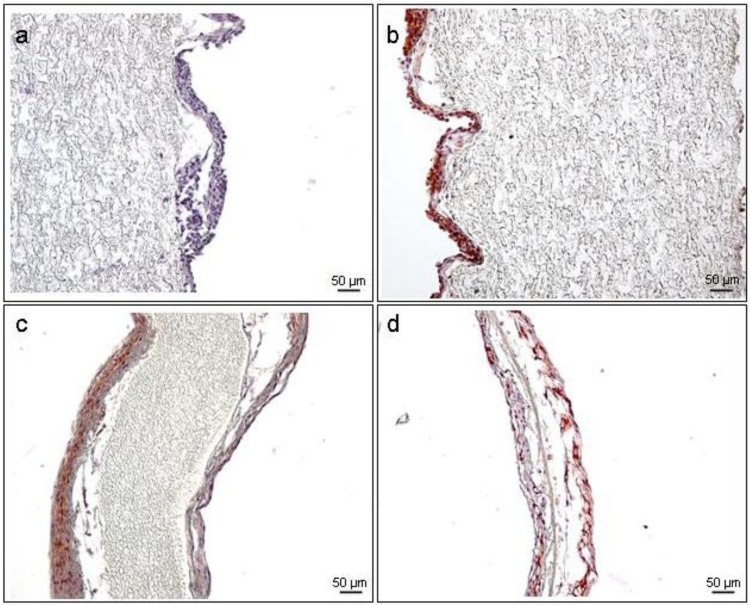
IHC analysis of seeded PUHV. Prior conditioning, seeded PUHV revealed a confluent coverage with FB (**a**) purple; and EC (**b**) brown. After conditioning PUHV also show a dense layer of FB (**a**) brown, arrows; and EC (**b**) brown, arrows. Cell nuclei were stained with hemalaun (**a**–**d**) purple. Scale bars = 50 µm (**a**–**d**).

Immunohistochemical analysis was carried out to compare fibroblast and endothelial layers prior and after conditioning. As shown in Figure 9, immunohistochemical analysis of PUHV prior conditioning revealed a positive staining for CD31 and TE-7, indicating a confluent coverage with FB (a, purple) and EC (b, brown). After conditioning, a multilayer of FB (c, brown) with an endothelial lining (d; brown) was observed. Cell nuclei were stained with hemalaun (a–d, purple).

## 3. Experimental Section

### 3.1. Construction of the Bioreactor

A three-dimensional model of the bioreactor was designed using CATIA V5R19 software (IndustrieHansa Consulting & Engineering GmbH, Munich, Germany). The bioreactor was divided in eight assembly groups (Table 1) and was manufactured in-house.

**Table 1:** Bioreactor assembly groups. The bioreactor was divided in eight assembly groups with several components: membrane carrier (**A**); valve (**B**); core unit (**C**); monitoring unit(**D**); actuator (**E**); eccentric (**F**); motor unit (**G**) and base unit (**H**).

Group	Description	Components
A	Membrane Carrier	2 aluminum coils, 4 fixing pins, 1 membrane, 1 seal ring
B	Valve	1 valve membrane, 4 guiding rings
C	Core Unit	1 housing, 1 air chamber, assembly A, assembly B, 1 lid, 1 Teflon^®^ support
D	Monitoring Unit	1 endoscope cylinder, glass plate, fixing screw
E	Actuator	1 cylinder, piston, 2 seal rings, cylinder lid
F	Eccentric	1 extender wheel, 1 piston rod, 2 securing pins, 2 plates
G	Motor Unit	2 stands, 1 motor housing, 1 motor
H	Base Unit	1 base plate, 1 reactor stand

A membrane carrier is designed to tighten the actuation membrane which is displacing the medium in the core unit. It consists of two aluminum coils with an outer diameter D = 102 mm, an inner diameter d = 70 mm and a height h = 8 mm. The membrane (D = 100 mm, thickness = 1 mm) is placed between the two coils. Four fixing pins tighten the aluminum coils and secure the membrane carrier against rotation. This way the attached seal ring is protected and a tight seal is guaranteed. The coils as well as the fixing pins are made of aluminum (Rau GmbH & Co.KG, Munich, Germany). Membrane and the seal ring consist of room temperature vulcanization (RTV) silicone (Sahlberg GmbH & Co.KG, Munich, Germany).

The membrane carrier, a valve and a cylindric air chamber (D = 120 mm, d = 70 mm, h = 60 mm) form the core unit. The valve consists of a silicone membrane (D = 72 mm, thickness = 1 mm) and four guiding rings (D = 10 mm, h = 5 mm) made of Teflon^®^ (Sahlberg GmbH & Co.KG, Munich, Germany). The valve is attached to the housing by four stainless steel screws (Keller & Kalmbach GmbH, Unterschleißheim, Germany; M6). The housing, made of acrylic glass (Sahlberg GmbH & Co.KG, Munich, Germany), can be described as a cylinder (D = 105 mm, h = 130 mm) divided in two chambers (d = 70 mm, h = 30 mm; d = 90 mm, h = 88 mm) by an offset. The barrier provides a fixation for a PUHV TS in the upper chamber and the valve in the lower chamber. The TS consist of a bottom plate (D = 48 mm, d = 27 mm, h = 13 mm) and a lid (D = 48 mm, d = 21 mm, h = 13 mm) with variable inserts connected by four bars (D = 6 mm, h = 55 mm). The chambers are connected by a MFDH (D = 27 mm) positioned in the center of the cylinder and eight smaller holes (D = 7 mm), circular arranged around the center. A rim (D = 102 mm) allows the placement of the membrane carrier. A horizontal bore hole (D = 15 mm, h = 23 mm) provides the ability to attach the actuator. The upper part of the core unit is sealed by a lid (D = 120 mm, h = 20 mm) made of acrylic glass with a centered bore hole (D = 18 mm, M27 thread) for endoscopic monitoring.

The monitoring unit is an assembly of an endoscope cylinder (D = 27 mm, d = 21 mm, h = 100 mm), a glass plate (Sarstedt AG & Co., Nümbrecht, Germany, D = 24 mm, h = 1 mm) and a fixing screw. The glass plate covers the lid hole and is secured by the endoscope cylinder. A horizontal thread (M6) allows securing the endoscope with the fixing screw.

The actuator consists of a cylinder, a piston, two seal rings and a cylinder lid. The cylinder (D = 45 mm, d = 40 mm, length l = 95 mm) provides six bores for fixation to the air chamber with stainless screws (Keller & Kalmbach GmbH, Unterschleißheim, Germany; M5). The inner diameter of the cylinder is reduced to 15 mm at the intake of the air chamber due to geometrical restrictions. The piston has a total length of 78 mm. The piston head (l = 16 mm, D = 28 mm) provides space for two seal rings. The piston shaft (l = 62 mm, D = 14 mm) provides a through hole (D = 6 mm) for the attachment of an eccentric. The cylinder lid (D = 40 mm, h = 4 mm) with a center bore (D = 16mm) provides space for the piston shaft. Cylinder, piston as well as cylinder lid are fabricated from aluminum.

The piston is connected to an eccentric (D = 56 mm, h = 6 mm) by a piston rod (l = 60 mm, width w = 10 mm, h = 6 mm) with a securing pin (D = 6 mm, l = 21 mm). To minimize the friction between the piston rod and the eccentric, a Teflon^®^ plate (D = 12 mm, d = 6 mm, h = 2 mm) was added interjacent. A thread (M3) assures a frictionless fixation to the motor. Piston rod and extender wheel are manufactured from aluminum. For the securing pins and plates, Teflon^®^ was chosen to minimize friction. The extender wheel is directly connected to the motor unit. The commercial available motor (RB-35GM; Modelcraft Inc., Blaine, WA, USA) with a copper coil and an iron shaft provides 60 revolutions per minute (rpm) and was connected to a speed regulator (H-Tronic GmbH, Hirschau, Germany) adjustable from 0 to 60 rpm. In order to avoid corrosion due to humidity, all metals are coated with an anti-corrosion layer. In addition, speed regulator and motor are placed in housings made of plastic (Sahlberg GmbH & Co.KG, Munich, Germany; l = 150 mm, w = 80 mm, h = 40 mm) and aluminum (Rau GmbH & Co.KG, Munich, Germany; D = 50 mm, l = 70 mm), respectively. 

The base unit consists of a rectangular base plate (l = 260 mm, w = 240 mm, h = 5 mm) and a cylindric reactor stand (D = 130 mm, h = 18 mm) with a countersink of 3.5 mm (D = 120 mm) to fit the air chamber. Base plate and reactor stand were manufactured from polyvinylchloride (PVC; Sahlberg GmbH & Co.KG, Munich, Germany); the air chamber was made of acrylic glass.

### 3.2. Sterilization of the Bioreactor

All bioreactor parts which are in contact with culture medium (group A-D) were disassembled and sterilized by formaldehyde deposition at 60–70 °C for 7 h. Medium samples were aseptically taken 5 days, 10 days and 15 days after bioreactor actuation in a standard incubator at 37 °C/5% CO_2_. Sterility was examined by conventional microbiological evaluation methods (Max-von-Pettenkofer-Institut für Hygiene und medizinische Mikrobiologie, University of Munich, Munich, Germany). In addition, the bioreactor was visually inspected and the air chamber was examined for traces of culture medium.

### 3.3. Seeding of PUHV

**Figure 10:**
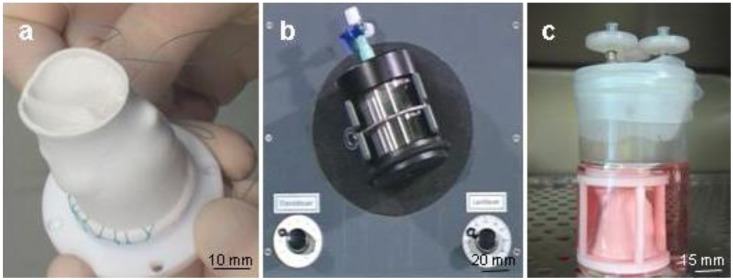
Seeding of PUHV. PUHV were sutured to the TS (**a**) and were consecutively seeded with human vascular fibroblasts and endothelial cells using a 3D-rotating bioreactor (**b**) for 24 h at 37 °C/5% CO_2_. After the dynamic seeding procedure, PUHV were statically cultivated for 6 days at 37 °C/5% CO_2 _ in a glass container (**c**). Scale bars: a = 10 mm, b = 20 mm, c = 15 mm.

The PUHV were sutured to the TS (a) and were seeded as previously described [33]. Briefly, PUHV were successively seeded with human vascular FB (1.5 × 10^6^/cm^2^) and EC (1.5 × 10^6^/cm^2^). For each cell type, a dynamic seeding procedure was performed for 24 h at 37 °C/5% CO_2_ using a 3D-rotating bioreactor (Figure 8a; running phase: 2.5 min; static phase: 30 min) followed by a static resting period of 6 d at 37 °C/5% CO_2 _in a glass container (Figure 10b). Medium was exchanged every 2–3 days.

### 3.4. Functionality Testing

An unseeded PUHV sutured to the TS was continuously preconditioned for 5 days using the newly developed bioreactor. The actuation unit (assembly groups E, F, G) was checked for proper operation in the humid climate of a conventional incubator at 37 °C/5% CO_2_ by monitoring of piston and eccentric movement. Furthermore, the actuation unit was examined for traces of abrasion and wear and the membranes were checked for ruptures. The quality of the images produced by the endoscope was evaluated. The endoscope was used to control the correct opening and closing of the PUHV during conditioning. The culture medium was visually examined on a regular basis to control the nutrient supply. For the analysis of the mechanical integrity and the conditioning efficiency of the cellular coating, seeded PUHV sutured to the TS was transferred under sterile conditions from the 3D-rotating seeding bioreactor to the newly developed pulsatile conditioning bioreactor and was continuously preconditioned at 750 mL/min for 2 days and 1,100 mL/min for 3 days. For analysis, samples of seeded PUHV were taken from the supravalvular, valvular and subvalvular region of the aortic wall as well as from the valvular leaflets. 

### 3.5. Scanning Electron Microscopy (SEM)

Samples from PUHV were fixed in 456 mL aqua bidest (Ampuwa, Fresenius Kabi Deutschland GmbH, Bad Homburg, Germany) supplemented with 0.75 mL 1 N hydrochloric acid (Titrisol, Merck KGaA, Darmstadt, Germany), 43.5 mL glutaraldehyde (Sigma-Aldrich Chemie GmbH, Steinheim, Germany) and 5.65 g sodium cacodylate trihydrate (Sigma-Aldrich Chemie GmbH, Steinheim, Germany) at 4 °C for 48 h. The fixed specimens were dehydrated in an afferent ethanol series (30%, 50%, 70% and 96% EtOH) and then placed in 100% acetone (Merck KGaA, Darmstadt, Germany). After critical point drying the samples were sputtered with gold for 180 s at 10–5 mbar and analyzed using a scanning electron microscope (Carl Zeiss Mikrolmaging GmBH, Göttingen, Germany).

### 3.6. Immunohistochemistry (IHC)

Immunohistochemical staining was performed to differentiate between FB and EC layers on the PUHV prior and after conditioning. Samples were fixed in 4% formaldehyde (Microcos GmbH, Garching, Germany) for 10 days at 4 °C and the stents were carefully removed. Paraffin-embedded specimens were sectioned at 10 µm and were stained for CD31 (0.09 µg/mL; Dianova GmbH, Hamburg, Germany) and TE-7 (2 µg/mL, Millipore Corporation BioScience Division, Temecula, CA, USA) using the HRP Detection System (Biozol GmbH, Eching, Germany) according to manufacturer’s protocol. Briefly, endogenous peroxidase activity was blocked using 0.12% H_2_O_2_ in PBS for 10 min at room temperature (RT). After incubation with the primary antibody overnight at 4 °C, the samples were incubated with the biotinylated link for 10 min at RT. The cells were then incubated with HRP Streptavidin-label for 10 min at RT, followed by AEC labeling (Vector Laboratories, Inc., Burlingame, CA, USA) for another 10 min at RT. Cell nuclei were stained with Mayer’s hemalaun (Merck KGaA, Darmstadt, Germany; 1:4 in PBS). Negative controls for non-specific binding of the biotinylated link were performed by exclusion of the primary antibody. Sections were analyzed using bright field microscopy (Carl Zeiss Mikrolmaging GmBH, Göttingen, Germany). 

## 4. Conclusions

In conclusion, we demonstrated the successful development of a pulsatile bioreactor for the conditioning of PUHV to shear stress. Functional testing showed that the bioreactor worked properly. This bioreactor means a large step towards long-life PUHV. These valves would not only be highly biocompatible but also provide the possibility to self-repair, which would be a significant advantage against all other synthetic heart valves. These advantages combined with a nearly endless availability render PUHVs highly attractive for further researches. With the bioreactor described above, a new type of conditioning bioreactors is introduced. The most important features of this reactor are an easy assembly, a sensitive regulation of stress and detailed monitoring combined in a compact design with high modularity. As a result of the introduction of a new step in tissue engineering, the preconditioning process, confluent and stable layers of autologous cells on synthetic or natural scaffold can be generated. This results in a gain of significance of tissue engineered heart valves in medical applications. The presented bioreactor was designed for PUHV-preconditioning. However, due to the modularity, the bioreactor system could be also used for multiple other applications. If the design is slightly readjusted, cardiovascular patches or vascular grafts could be perfused in this system. For these applications, only customized core units have to be designed. The actuation unit as well as the monitoring unit do not have to be changed and can be used for all designs, leading to a cost- and time-effective design and manufacturing process of new customized reactors. 
